# What Will the Profession Gain by Combinations?

**Published:** 1914-01-03

**Authors:** F. L. Nicholls

**Affiliations:** Fulbourn


### THE EDITOR'S LETTER-BOX.

### What Will the Profession Gain by Combinations?

*To the Editor of* THE HOSPITAL.

SIR,—At the present time we see the profession divided into practically two parties; these are busy forming themselves into associations for self-defence. After what one has seen in the recent past is one not justified in doubting the value of associations for such a purpose? A little more than twelve months ago the profession was believed to be in an impregnable position; practically all its members had signed a pledge and the public believed them to be a class of men who would keep their word. A little pressure was put on, a stampede took place, and the majority showed themselves careless of their pledged word; the reason given being that the authorities were prepared to send what in the picturesque language of the trade unionist is called a "blackleg" or "scab" to take their work. Now it must have been clear to those men practising in thick industrial districts, if they had given it a moment's thought, that as their practice was wholly or almost wholly contract they would be unable to keep their pledge unless they gave up their work, and the signing of the pledge by them could only have been a species of bluff. The point, therefore, is that if the past apparently very favourable stand broke down through this class of men, can a better stand be expected in the future? The answer to this must be in the negative, for many men are now doing far better financially by "panel" work than they ever did before. It would, therefore, seem as if the only chance for making a stand in the future would be by local combinations. Here, again, I see little hope, for men will always be got to take the work of their fellows. The future of the profession, therefore, must depend upon the individuality, self-respect, and independence of the superior type of men, who have already shown their ability to obtain for themselves a class of practice so as to make them indifferent to or independent of contract work. It is this class of men who will keep the profession in the respect of the public and prevent it from sinking as a body to the position now held by "panel" men.—I am, Sir, etc.,

Fulbourn, December 22, 1913. F. L. NICHOLLS.

